# Beyond the blueprint: decoding calmodulinopathy—a case report showcasing the utility of multifaceted treatments

**DOI:** 10.1093/ehjcr/ytaf140

**Published:** 2025-04-01

**Authors:** Saikiran Kakarla, Madhusoodanan Jalaja Aswathy, Madhusoodanan Pillai Sreelekshmi, Machilakath Panangandi Shabeer, Narayanan Namboodiri

**Affiliations:** Department of Cardiology, Sree Chitra Tirunal Institute for Medical Sciences and Technology, Medical College Junction, Thiruvananthapuram, Kerala 695011, India; Department of Cardiology, Sree Chitra Tirunal Institute for Medical Sciences and Technology, Medical College Junction, Thiruvananthapuram, Kerala 695011, India; Department of Cardiology, Sree Chitra Tirunal Institute for Medical Sciences and Technology, Medical College Junction, Thiruvananthapuram, Kerala 695011, India; Department of Pediatrics, IQRAA International Hospital and Research Centre, Malaparamba, Calicut, Kerala 673009, India; Department of Cardiology, Sree Chitra Tirunal Institute for Medical Sciences and Technology, Medical College Junction, Thiruvananthapuram, Kerala 695011, India

**Keywords:** Calmodulinopathy, Calmodulin, Long QT syndrome, Mexiletine, Case report

## Abstract

**Background:**

Calmodulinopathies are adrenergically-induced life-threatening arrhythmias. Available therapies are disquietingly insufficient, especially for CALM-LQTS (calmodulinopathy-associated long QT syndrome). This case report illustrates a novel mutation in CALM-LQTS and its response to multimodality treatment strategies.

**Case summary:**

The proband was the first child born to a nonconsanguineous Indian couple, a 26-year-old woman and a 30-year-old man. The child was delivered prematurely, and at birth, a functional 2:1 atrioventricular block was noted with sinus bradycardia with a corrected QT by Bazzet’s of 716 ms. Clinical exome sequencing of the proband revealed a novel missense variant c.287A>G in exon 5 of the CALM3 gene in a heterozygous state, resulting in an Asp96Gly change. The OMIM phenotype associated with it is long QT syndrome 16 (#618782). Despite receiving a dose of 4.5 mg/kg/day of propranolol, the child still had a persistent long QTc. Mexiletine was started at the trial dose of 1.5 mg/kg/day, and after 1 h, QTc was reduced to 507 ms from 560 ms. After a left-cardiac sympathectomy, he remains asymptomatic after 1.3 years of follow-up with a QTc value of 490 ms.

**Discussion:**

CALM3 pathogenic variants are gain-of-function variants mainly affecting amino acids residing in the Ca2-binding loops. Earlier data suggested the role of the Na_v_1.5 channel in leading to persistent Na^+^ leaks resulting in LQTS. However, they only focused on LQTS-CALM1 and CALM2 models and did not include CALM3-related genes. Despite similarities, the precise impact of CaM on Na_v_1.5 channels still needs to be defined as Ca_V_1.2. The exact role of mexiletine is not fully understood.

**Learning points**

- Calmodulinopathies are life-threatening arrhythmias with varying phenotypical presentations; the most common among them was long QT syndrome, which distinctly has perinatal presentation, functional 2:1 AV block and *de novo* pathogenic mutations.
- This case report discusses the various treatment options available for a novel CALM-3 mutant and examines the effect of mexiletine on the QTc. Mexiletine may have a mutation-specific role. Further molecular and electrophysiological studies are needed to determine its effectiveness.

## Introduction

Calmodulinopathies are adrenergically-induced life-threatening arrhythmias. Though therapies are available, prompt diagnosis and early use of combination therapy, especially for CALM-LQTS (calmodulinopathy-associated long QT syndrome), are important hurdles.^1,2^ Considering the most extensive data, it is advisable to contemplate combination therapy involving medications, left cardiac sympathetic denervation (LCSD), and cardioverter-defibrillator devices.^1^ This case report illustrates a novel variant in CALM-LQTS and its response to multimodality treatment strategies.

## Summary figure

**Figure:**
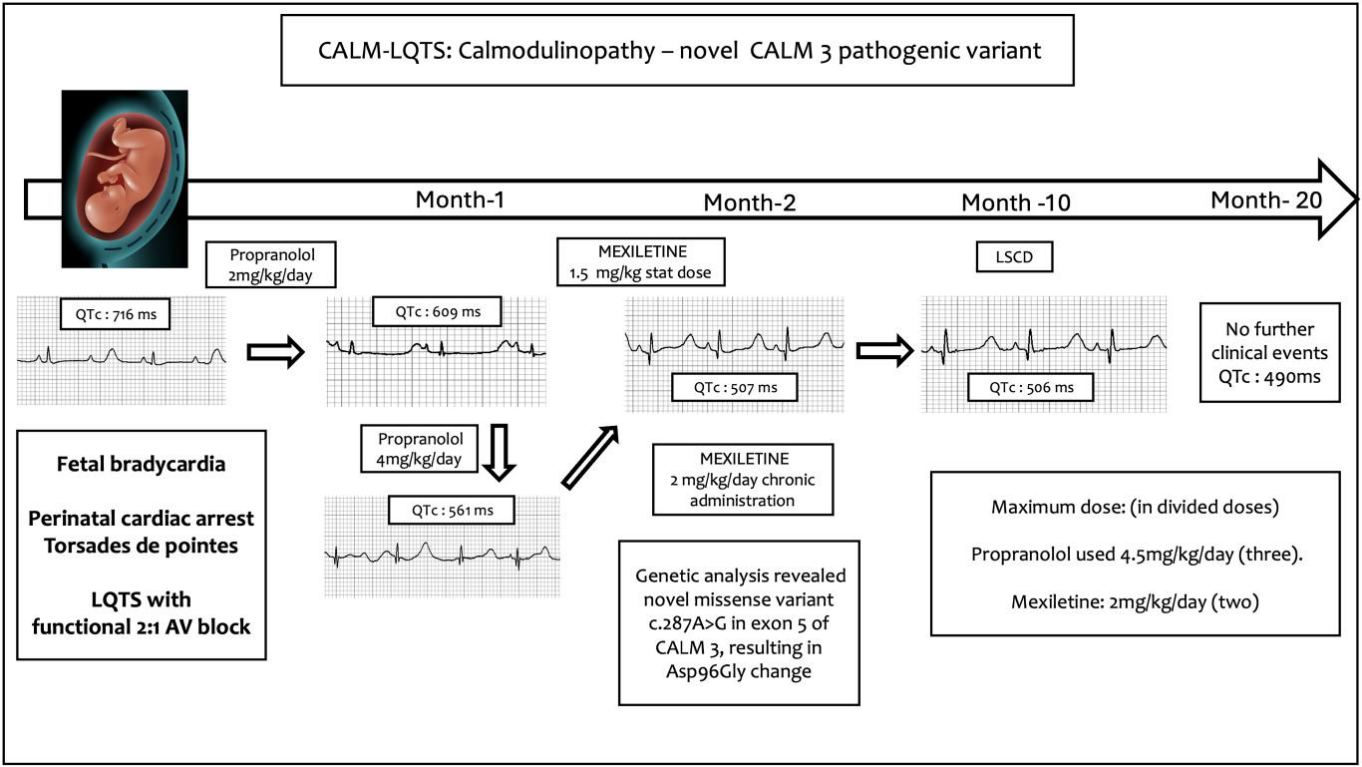


## Case presentation

**Figure 1:**
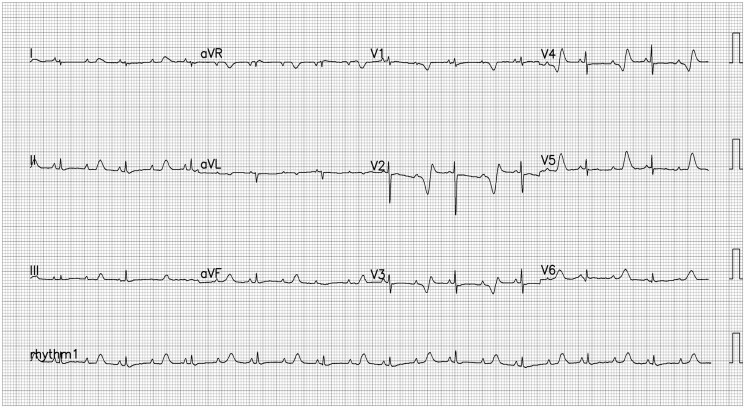
Surface 12-lead ECG showing markedly prolonged QTc with functional 2:1 atrioventricular block.

**Figure 2:**
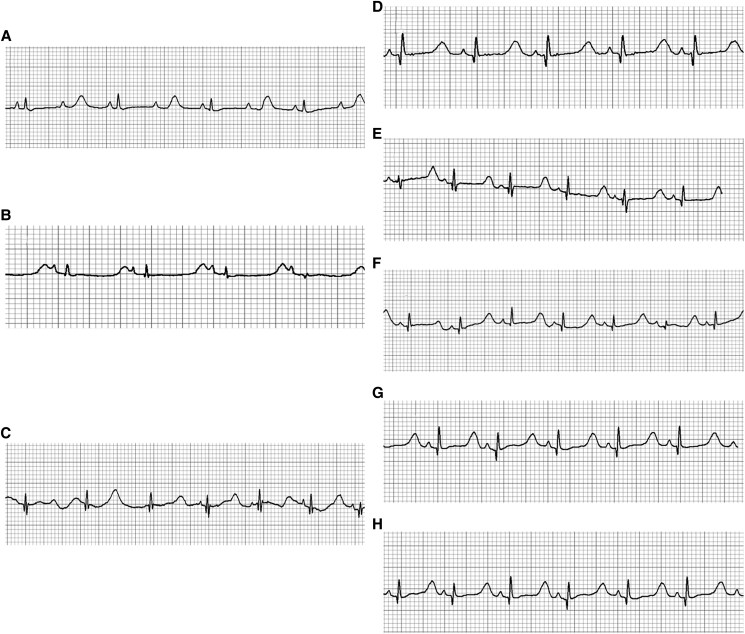
Surface ECG of the reported case (QT interval after Bazzet’s correction). (*A*) Baseline ECG at Day 1 of life with 2:1 AV block and long QT syndrome (QTc—716 ms); (*B*) disappearance of 2:1 AV block (QTc—609 ms); (*C*) ECG taken after tolerable doses of propranolol intake and before mexiletine intake (QTc—561 ms); (*D*) ECG after 1 h of mexiletine intake (QTc—507 ms); (*E*) after 3 h of mexiletine intake, QTc—503 ms; (*F*) after 6 h of mexiletine intake, QTc—503 ms; (*G*) after 8 h, QTc—507 ms; (*H*) after 12 h, QTc—534 ms.

The proband was the first child born to a nonconsanguineous Indian couple, a 26-year-old woman and a 30-year-old man. Mother’s pregnancy was uneventful, and there were no sudden cardiac deaths in the family. At 30 weeks, foetal ultrasonography found bradycardia with no structural heart disease. The child was delivered prematurely at 30 weeks via emergency caesarean section due to abnormal foetal Doppler. At birth, a functional 2:1 atrioventricular block was noted with sinus bradycardia with a corrected QT by Bazzet’s of 716 ms (*Figure 1*). Child had cardiac arrest, likely due to Torsades de pointes from bradycardia and was promptly resuscitated and started on oral propranolol for QT prolongation. No ECG tracings were available. The 2:1 AV block was resolved, but the QTc was 609 ms. (*Figure 2*). Parents’ ECGs and QT intervals are normal.

Clinical exome sequencing of the proband revealed a novel missense variant c.287A>G in exon 5 of the CALM3 gene in a heterozygous state, resulting in an Asp96Gly change. In silico analysis, the variant was predicted to be disease-causing. The reported variant has been absent in the genetic population databases so far. The variant was a *de novo* pathogenic variant and not found in either parent upon Sanger sequencing. The OMIM phenotype associated with it is long QT syndrome 16 (#618782).

Later, despite the optimum weight-adjusted doses of propranolol (4.5 mg/kg/day in three divided doses), the child had persistent long QTc of 561 ms. Hence, after a shared clinical decision, mexiletine was started with a leading dose of 1.5 mg/kg/day in two divided doses as a part of the therapeutic trial (no further loading doses were administered). After 1 h of ingestion, QTc was reduced to 507 ms from 561 ms. The heart rates before and after mexiletine were 96 and 108 b.p.m., respectively, during the assessment of QTc. This decrement was over and above the effect of propranolol (*Figure 2*). QTc remained stable at around 507 ms, and after 12 h of drug intake, QTc increased to 534 ms. On follow-up, an attempt to increase the dose of mexiletine to 4 mg/kg/day resulted in symptoms of neuronal irritability. Hence, the child was maintained with tolerable doses of mexiletine (2 mg/kg/day in two divided doses) and propranolol (4.5 mg/kg/day in three divided doses) for up to 10 months of life.

Given the reported poor survival with drugs alone in the literature, after a shared clinical decision, with informed consent, by left posterolateral thoracotomy approach, LCSD was done at 10 months of life. The child weighed 7.5 kg and was 69 cm long during the procedure. The child was intubated with a single-lumen endotracheal tube, and the lung was retracted to expose the dorsal ganglia. The lower half of the left stellate ganglion and thoracic ganglia from T2–T4 were excised. No anatomical variation in the sympathetic ganglia was noted. QTc was 507 ms before LCSD and remained at 503 ms after. At six months of follow-up, QTc was 490 ms. Currently, at 20 months of life, he remains asymptomatic (a timeline of case findings is outlined in the *Summary figure*).

## Discussion

Calmodulinopathies encompass a diverse yet complex phenotypic spectrum of channelopathies, which are long QT syndrome (LQTS) (49%) and catecholaminergic polymorphic ventricular tachycardia (CPVT) (28%).^1^ As per the international calmodulinopathy registry, any structural heart disease like cardiomyopathy or congenital heart defects is present in 30% of patients; among them, 78% were CALM-LQTS subsets.^2^ Other less common phenotypes are LQTS-CPVT overlap, idiopathic ventricular fibrillation, sudden unexplained deaths, and neurological impairment. The distinctive features of this genetic channelopathy, particularly CALM-LQTS, are *de novo* pathogenic variants with often a perinatal presentation and associated functional 2:1 AV block.^1^

So far, 10 CALM3 pathogenic/likely pathogenic LQTS variants (and one variant of unknown significance: VUS) have been documented in the literature.^1–4^ CALM3 pathogenic variants are gain-of-function variants mainly affecting amino acids residing in the Ca2-binding loops (EF-hands III and IV). Functional characterization of several LQTS-associated calmodulin (CaM) pathogenic variants has shown that they mostly affect its Ca^+2^-binding affinity, consequently impairing the Ca2-dependent inactivation (CDI) of the L-type Ca^+2^ channel (Ca_V_1.2). In contrast, the other ion channels regulated by CaM are less consistently affected.^5,6^

Earlier data suggested the role of CaM in regulating the cardiac Na channel (Na_v_1.5) inactivation, and a specific defect in Na_v_1.5 inactivation (increased persistent Na current) occurs in LQTS.^7^ Na_v_1.5 channels interact with CaM via an IQ motif on their C-terminus, independent of Ca^2+^, and play a crucial role in arrhythmogenesis.^8^ Unlike Ca_V_1.2 channels, this interaction doesn’t trigger conformational changes. Disrupting this site boosts persistent Na^+^ current, indicating CaM’s role in stabilizing inactivation. CaM binding may hinder direct Ca^2+^ modulation, causing a negative shift in Na_v_1.5 channels’ steady-state inactivation curve and affecting their function.^5,6,8–10^ Previous studies using genetically engineered co-expression models of LQTS-CaM pathogenic variants and Na_v_1.5 have revealed the inconsistent role of Na_v_1.5 channels in pathogenesis. Unfortunately, Yin *et al*.’s study mainly explored LQTS-CALM1 and CALM2 models (CALM1-related genes with amino acid substitutions N54I, N98S, D130G, F142L, and D96V of CALM2 genes), with limited attention to the role of CALM3-related genes. This overlook limits the comprehensive understanding of the subject and highlights the need for further research on CALM3-related genes.^11,12^ While CaM’s interaction with Na_v_1.5 channels shows similarities to its interaction with Ca_V_1.2, its specific mechanisms and consequences require further clarification.

Similarly, in another family reported by Wren *et al*.,^3^ a novel mutant of CALM3 with a milder clinical phenotype alluded to a modest reduction of CDI of the L-type calcium channel and augmentation of Kv7.1 (Iks).

## Calmodulinopathy and left cardiac sympathetic denervation

Left cardiac sympathetic denervation increases the threshold for VF by decreasing the release of norepinephrine and reflexively increasing cardiac vagal efferent activity.

Single lung ventilation (SLV) during the procedure is particularly challenging in infants and small children, as their lungs are soft and easily compressible. The residual volume is closer to functional residual capacity and can easily reduce lung compliance, increasing airway closure even during tidal ventilation. Optimal SLV strategies include single-lumen endotracheal tubes, balloon-tipped bronchial blockers, double-lumen endobronchial tubes, uninvent tubes, or double-lumen tubes.^13^

Many previous studies showed variable QT shortening after LCSD, which is subjective to multiple factors. In a recent study, in patients with particularly prolonged QTc > 500 ms, half of their QT was reduced by ≥30 ms (mean of 64 ms ± 24 ms) by LCSD.^14^ This change is often variably seen before discharge but manifests at six months of follow-up. Patients whose QTc is reduced to <500 ms after LCSD have a better prognosis. Genotype may not influence the QT shortening effect of LSCD in LQT1 and LQT2 subsets. A greater reduction of the QTc value was noted in high-risk subsets (such as CALM/CACNA1C/Jervell and Lange-Nielson syndrome) with QTc > 550 ms before LSCD. This suggests that LSCD may be particularly effective in reducing QTc in very high-risk subsets.^14^

In our index case, there was a reduction in QTc after a leading dose of mexiletine (1.5 mg/kg/day) administration, and the response was over and above the beta blockers, similar to a case from the International Calmodulinopathy Registry.^1^ The noticeable decrease in QTc, even with a reduced dosage of mexiletine, might imply the presence of genetic polymorphisms in the CYP2D6 gene. These polymorphisms influence mexiletine metabolism and may lead to elevated plasma levels. This suggests that mexiletine may have a pathogenic variant-specific role in CALM3 subsets. Verapamil usage was considered unsafe for young children and was not attempted. It may be an option for older children.^15^ The QTc interval immediately after LCSD was similar to the pre-operative values but was reduced at six months follow-up.

## Conclusion

This case report demonstrates the use of various modes of treatment in a novel CALM3 pathogenic variant. The exact role of mexiletine is not yet fully understood in CALM3 subsets; hence, more in-depth electrophysiological and molecular studies, particularly focusing on CALM3 pathogenic variant models, are required to determine the drug’s significance.

## Data Availability

The data underlying this article will be available upon requesting the corresponding author.

## Lead author biography

The lead author is an assistant professor in the Department of Cardiology, Sree Chitra Tirunal Institute for Medical Sciences and Technology, Thiruvananthapuram, specialized in cardiac electrophysiology and pacing.
