# Correction to: Beyond the blueprint: decoding calmodulinopathy—a case report showcasing the utility of multifaceted treatments

**DOI:** 10.1093/ehjcr/ytaf277

**Published:** 2025-06-27

This is a correction to: Saikiran Kakarla, Madhusoodanan Jalaja Aswathy, Madhusoodanan Pillai Sreelekshmi, Machilakath Panangandi Shabeer, Narayanan Namboodiri, Beyond the blueprint: decoding calmodulinopathy—a case report showcasing the utility of multifaceted treatments, *European Heart Journal - Case Reports*, Volume 9, Issue 4, April 2025, ytaf140, https://doi.org/10.1093/ehjcr/ytaf140

In the originally published version of this manuscript, the affiliations for authors Machilakath Panangandi Shabeer and Narayanan Namboodiri were inadvertently swapped. Machilakath Panangandi Shabeer is affiliated with the Department of Pediatrics, IQRAA International Hospital and Research Centre. Narayanan Namboodiri is affiliated with the Department of Cardiology, Sree Chitra Tirunal Institute for Medical Sciences and Technology. This error has been corrected.

